# Dataset of Arabic spam and ham tweets

**DOI:** 10.1016/j.dib.2023.109904

**Published:** 2023-12-05

**Authors:** Sanaa Kaddoura, Safaa Henno

**Affiliations:** Zayed University, Abu Dhabi, UAE

**Keywords:** Twitter, Labelled data, Classification, Machine learning, Deep learning, Cybersecurity, Social network analysis

## Abstract

This data article provides a dataset of 132421 posts and their corresponding information collected from Twitter social media. The data has two classes, ham or spam, where ham indicates non-spam clean tweets. The main target of this dataset is to study a way to classify whether a post is a spam or not automatically. The data is in Arabic language only, which makes the data essential to the researchers in Arabic natural language processing (NLP) due to the lack of resources in this language. The data is made publicly available to allow researchers to use it as a benchmark for their research in Arabic NLP. The dataset was collected using the Twitter REST API between January 27, 2021, and March 10, 2021. An ad-hoc crawler was constructed using Python programming language to collect the data. Many scientists and researchers will benefit from this dataset in the domain of cybersecurity, NLP, data science and social networking analysis.

Specifications Table

**Table:** 

Subject	*Data Science*
Specific subject area	The dataset contains tweets to help data scientists to create machine learning models to classify tweets as either spam or ham automatically. It is useful for data science.
Data format	Raw
Type of data	Table
Data collection	Ham tweets were collected from famous verified accounts (e.g., Arabiya, emaratalyoum, and skynewsarabia). Spam tweets were collected by querying Twitter using specific Arabic spam keywords. Then, the top 10 accounts with high spam percentages were crawled. Then, the list was inspected to remove ham tweets manually. All duplicate tweets were removed for both ham and spam. The spam data may contain inappropriate words because they are spam.
Data source location	The dataset includes all the geotagged and non-geotagged tweets posted in Arabic from any country and location. For ham tweets, only verified accounts were used (Al Arabiya, Al Hadath, Emarat Alyoum, and Sky News Arabia).
Data accessibility	Repository name: Mendeley Data identification number: 10.17632/86x733xkb8.1 Kaddoura, Sanaa; Henno, Safaa (2023), “Dataset of Arabic Spam and Ham Tweets”, Mendeley Data, V1, doi: 10.17632/86x733xkb8.1 Direct URL to data: https://data.mendeley.com/datasets/86x733xkb8/2 The spam data in the dataset are anonymized so that no information about the user can be identified. The ham data is listed with its sources. The spam data may contain inappropriate words because they are spam.
Related research article	S. Kaddoura, S.A., Alex, M. Itani, S. Henno, A. AlNashash, D.J. Hemanth. Arabic spam tweets classification using deep learning. Neural Computing and Applications. 2023 Apr 29:1-4. https://doi.org/10.1007/s00521-023-08614-w

## 1 Value of The Data

• Arabic is considered a low-resource language due to the lack of datasets. Posting this dataset for public use contributes to Arabic natural language processing research.
• The dataset allows researchers to develop and evaluate spam detection algorithms for the Arabic language. The dataset is valuable for training machine learning models to create effective classifiers that can automatically identify and filter out spam tweets from social media posts. This is crucial to the trustworthiness of information on social media platforms.
• Data scientists can utilize this dataset to explore novel techniques for spam detection, develop text classification algorithms, investigate user behaviour, and analyze social dynamics. In addition, researchers in the cybersecurity industry can leverage this dataset to develop robust spam detection systems, improve content moderation processes, and enhance user experience.
• The dataset is a valuable resource for developing and refining machine learning-based algorithms. Various machine learning algorithms, such as decision trees, support vector machines, neural networks, or ensemble methods, can be applied to train models for spam detection or text classification. By comparing the performance of various algorithms on the dataset, researchers can identify the most effective techniques and explore novel approaches. Researchers can use the dataset for pretraining models on a large corpus of tweets, including spam and non-spam, which can capture the contextual understanding of the Arabic language and improve the performance of downstream tasks. The pre-trained models can be fine-tuned for specific applications or domains.
• The e-commerce sector heavily relies on social media platforms to gauge consumer sentiments, trends, and preferences. This dataset can be utilized to detect spam in customer feedback. Detecting spam and fraudulent reviews is crucial for maintaining the integrity of customer feedback. This dataset can be used to train models to identify fake reviews and protect consumers from misleading information. This dataset can be used to support the educational sector. It is crucial to maintain the quality of content on educational websites. Educational discussions on social media should be focused on meaningful and informative content. Spam posts can clutter the platform with irrelevant and low-quality material, making it difficult for users to find valuable educational content. Moreover, an educational platform cluttered with spam can diminish the credibility of these institutions and experts, making it harder for genuine educational content to stand out.
• Speaking about the political sector, this dataset can be used for preserving authentic dialogues. Politics involves discussions of critical societal issues, policies, and viewpoints. Spammers can disrupt these conversations by flooding platforms with irrelevant content, making it harder for users to engage in meaningful and genuine discussions. The dataset can contribute to the health sector as well. The health sector relies on accurate and reliable information. Spammers can disseminate false medical advice, misleading remedies, promoting unsafe or fraudulent products and unverified treatments, potentially risking people's health and lives. Effective spam detection helps prevent the spread of such misinformation. In the technology sector, spam detection helps prevent the spread of malware, viruses, and malicious software through spammy links or attachments. It also aids in identifying fake tech support services that might attempt to exploit users. So, this dataset can contribute to building a machine-learning-based model for this purpose.

## 2 Background

The dataset is related to the original article in [1]. Due to the lack of availability of datasets in Arabic, it was essential to collect a new dataset and create an ad-hoc crawler to create a deep-learning model for classifying spam tweets. The increase in the presence of malicious content on social media platforms is a motivation for this work. For the English language, detecting spam content has achieved a mature status in research. However, the research still needs much effort for other low-resource languages, such as Arabic. Spam tweets pose a significant challenge for users, as they can spread misinformation and post inappropriate content, which will negatively affect the user experience who are using online platforms almost daily. Consequently, there is a need for automated techniques to detect and classify such content. The availability of such a dataset encourages the exploration of novel approaches, feature engineering, and model architectures, ultimately leading to improving spam detection systems and enhancing the overall user experience on social media platforms.

## 3 Data Description

The dataset contains one .csv file. Table 1 contains the data count of the total tweets, spam, and ham tweets. There are 1941 spam tweets and 11299 ham tweets. In total, the tweets are 13240.

**Table 1:** Data count.

Tweets	Count
Spam	1941
Ham	11299
Total	13240

Table 2 presents a description of each column name in the dataset. Some column data were cleaned for the spam tweets to keep the user's identity anonymized for privacy issues. These columns are User Name, Location, Replied Tweet ID, Replied Tweet User ID, Replied Tweet, User Name, and Coordinates.

**Table 2:** Description of each column in the dataset.

Column name	Description
Date	The date when the tweet was posted.
Time	The time when the tweet was posted.
Date Time	The combination of date and time of the tweet post.
URL	The URL associated with the tweet, if any.
Tweet Text	The original text of the tweet, which may include hashtags, mentions, and other content.
Cleaned Text	The processed and cleaned version of the tweet text, with unnecessary characters and noise removed.
User Name	The username of the Twitter account that posted the tweet.
Location	The location mentioned or associated with the tweet if provided by the user.
Replied Tweet ID	The unique identifier of the tweet to which the current tweet is a reply, if applicable.
Replied Tweet User ID	The unique identifier of the user who posted the tweet being replied to, if applicable.
Replied Tweet User name	The username of the user who posted the tweet being replied to, if applicable.
Coordinates	The geographic coordinates associated with the tweet, if provided by the user.
Retweet Count	The number of times other users have retweeted the tweet.
Favorite Count	The number of times the tweet has been marked as a favorite by other users.
Favorited	A binary indicator (True or False) indicates whether the tweet has been favorited by the user who posted it.
Label	A label indicating whether the tweet is categorized as “spam” or “ham” (non-spam).

The decision to remove these columns from the dataset was driven by a paramount concern for user privacy, ethical considerations, and the necessity to adhere to stringent data protection guidelines in the Mendeley Data repository. In their policy [14], Mendeley Data states that data should not contain sensitive information (for example, but not limited to exact names, dates of birth, etc.). According to their 4.4.7 of Terms, data must be suitably anonymized wherever appropriate" [14]. From a privacy perspective, excluding these columns aims to safeguard the identities of individuals who contributed to the dataset, ensuring that their personal information remains confidential and shielded from potential misuse. User Name and Location, for instance, can often reveal sensitive details about an individual's identity and location, thus necessitating their removal to prevent potential identification.

Similarly, the removal of Replied Tweet ID, Replied Tweet User ID and Replied Tweet content was crucial to prevent potential traceability back to specific users or their interactions, maintaining the anonymity of user engagement. Moreover, excluding Coordinates was pivotal in preventing the inadvertent exposure of user geolocation information. By considering these factors and proactively removing these columns, the ethical integrity of our research was upheld while ensuring that the data remains analytically valuable and ethically responsible.

The Tweet Text field is the original text of the tweet. Since this dataset contains spam text, it is normal to see inappropriate text in this section. The goal of this work is to contribute to stopping such text on public social media platforms. The Cleaned Text column contains the tweet text after preprocessing and removing unwanted characters that may not affect the analysis stage.

Table 3 presents sample tweets from the dataset. The first column is the actual tweet in the dataset, whereas the second column is its English translation. The third column is the label of the Arabic tweet in the dataset, whether ham or spam tweet.

**Table 3:** Sample tweets and their English translation.

Arabic tweet	English translation	Label
حكم يتصدى لكرة في طريقها لمرمى في لقطة كوميدية نادرة منصات	A referee tackles a ball on its way to a goal in a rare comedy clip	Ham
شحنه جديده من لقاح كورونا الصيني تصل مطار القاهره	A new shipment of Chinese Corona vaccine arrives at Cairo Airport	Ham
وعليكم السلام ورحمة اله عندي مجموعه تنحيف لأطفال عباره عن مكمل غذائي يعمل على تسريع عملية حرق الدهون تعالي خاص بعطي لك حل	May the peace and mercy of God be upon you. I have a slimming kit for children. It is a nutritional supplement that accelerates the fat-burning process. Come on, I will give you a solution.	Spam
صوروا التغريدة قبل الحذف	Tweet before deletion	Spam

Although the utilization of various categories of spam tweets, such as advertisements, false information, and malicious content, resulted in high spam data samples, spammers often post spam tweets multiple times. As a result, these tweets contain several duplicates, urging the application of preprocessing to eliminate these duplicates. While the available dataset is sufficient for training deep learning models, it is valuable to augment it to increase the diversity and quality of data. Data augmentation [9], particularly, helps to balance the number of spam and ham tweets. However, augmenting Arabic text data in natural language processing presents challenges due to the complex nature of language. During the augmentation process, stop words are excluded, and data augmentation is applied to content terms only. When the dataset was used in [2], data augmentation was applied to the text after extracting the numerical feature. This technique was applied just before machine learning. These techniques apply K-nearest neighbor to perform oversampling for the majority class, support vector machine, or consider the density distribution.

The augmentation of data can be done through many other techniques such as the following:

• Synonym Replacement: where words within the sentence are substituted based on their meaning.
• Contextual augmentation: where words within the sentence are substituted while keeping the context.
• Character Augmentation: where random characters are chosen and replaced by another one, or two characters within the word are swapped.
• Back translation: where the sentence is translated into another language, such as English, and then translated back to Arabic.
• Random Deletion: where a random word is chosen and deleted from the sentence.
• Random Swap: where two words are chosen and swapped.
• Random Insertion: where random words are inserted into a sentence yield.
• Tense Alteration: where the tenses of the verbs are changed while keeping the same sentence.
• Masking: where words in a sentence are replaced with a distinct token.

Context augmentation is employed to augment the spam sample in the spam and ham tweet dataset and produce another version of the dataset for researchers to use. This type of augmentation is used due to the limitations of the other techniques. Applying random deletion, random swap, and masking might result in meaningless sentences and the absence of information. Altering the verb tenses while keeping the same sentence will create duplicates of spam messages. Although back translation proves its effectiveness across multiple languages, it may encounter limitations due to complexities when applied to Arabic. Character augmentation might result in unknown words. This has made contextual augmentation the most effective approach for Arabic text. To apply contextual augmentation, the transformer model, BERT, is utilized. Through this strategy, data augmentation effectively reduces overfitting and bias within machine learning. Furthermore, the augmentation of spam tweets aids in addressing the challenges posed by class imbalance. The augmented dataset specifications are now available in Table 4.

**Table 4:** Augmented dataset.

	Count
Spam	15128
Ham	11030
Total	26158

## 4 Experimental Design, Materials and Methods

The dataset was collected using the Twitter REST API between January 27, 2021, and March 10, 2021 [2]. In order to collect the data legally, a Twitter developer's account was created after describing to the Twitter platform the purpose and authenticity of the research. The following credential keys were input to the Python-based ad-hoc crawler in order to start collecting the data:

• OAuth access token secret
• consumer key
• OAuth access token
• consumer secret

To facilitate the extraction of tweets, the ad-hoc crawler has a process of sending queries to Twitter using predefined search terms. This approach allowed for targeted retrieval of relevant data from the platform. The collection of ham tweets representing non-spam or legitimate tweets was specifically obtained from a set of reputable Twitter accounts. These accounts, namely Al Arabiya [3], Al Hadath [4], Emarat Alyoum [5], and Sky News Arabia [6], are widely recognized as prominent news sources on the platform. Given their established reputation and credibility, it is assumed that all the posts from these accounts are carefully monitored, ensuring the absence of any spam tweets. Consequently, any tweet gathered from these verified accounts is categorized as a ham tweet, contributing to the legitimate data collection for analysis purposes.

Spam tweets were collected by querying Twitter using specific Arabic spam keywords and hashtags. The keywords and hashtags were selected based on the trends in Arabic hashtags throughout the data collection period, in addition to Arabic spam keywords extracted from [7]. Spam data potentially involves multiple categories, such as advertisements, false information, malicious content, and other data types incompatible with the designated hashtag theme. These categories specify the choice of spam keywords. For instance, the data was collected during the COVID-19 pandemic, so the hashtag #كورونا was adopted. However, some tweets that include this hashtag are not related to the pandemic discourse, instead propagating unsolicited advertisements for unverified weight-altering medications, often originating from anonymous or non-expert sources. These instances are recognized as spam data. Table 5 shows some of the keywords and hashtag lists used for data collection. The terms in Table 5 are used as both keywords and hashtags. The tweet was collected if these terms appear in the hashtag or as a keyword in the tweet.

**Table 5:** Spam keywords and hashtags.

Spam keywords and hashtags	English translation
الأسطورة	The Legend
كورونا	Coronavirus
ماجيستير عن بعد	Distance Master's
كوبون خصم	Discount Coupon
مباشر مباريات كرة قدم	Live Football Matches
نتفاكس	Netflix
بتعاني من زيادة الوزن والكرش او الترهلات	Suffering from Weight Gain or Belly Fat and Sagging
بكالوريوس للبيع	Bachelor's Degree for Sale
جامعية للبيع	University Degree for Sale
وفاة الفنان	Death of the Artist
رابط بث مباشر مشاهدة الأهل	Live Stream Link for Watching the Family
الفوركس	Forex
تحليل شخصية	Personality Analysis
قبل الحذف	Before Deletion
ايلتس معتمدة	Accredited IELTS
بيع متابعين	Sell Followers
بيع شهادات	Sell Certificates
بكالوريوس معتمدة	Accredited Bachelor's
معتمدة للبيع	Accredited for Sale
زيادة عدد المشاهدين	Increase Viewership
تحليل الشخصية	Personality Analysis
راحتي معصيتي	My Pleasure is My Sin
سناب عثمان	Snapchat Osman
سكس	Sexual Content
شاهد بالفيديو	Watch in the Video
التقطيع	The Cutting
رابط زيادة المتابعين	Link to Increase Followers

The top 10 accounts with high spam percentages were selected, and all the tweets on their timelines were collected. The collected spam tweets list was inspected manually to check if it contained any ham tweets. In case any ham tweets exist, it was removed and excluded from the dataset. The paper's two authors revised the collected spam tweets and marked any ham tweets. If both authors agree it is a ham tweet, it is directly removed. If one of them says it is spam and the other says it is ham, another professional was asked to give his opinion. Then, the tweet was marked according to the majority of opinions.

Both ham and spam tweets and their corresponding attributes were merged in one file to form the dataset. Also, duplicate tweets were removed. So, the dataset contains unique tweets only.

The tweet text was cleaned during the preprocessing stage. All tweets that contain characters other than the Arabic language characters were excluded from the dataset. The following list of characters was removed upon preprocessing of the tweet:

• Characters (@ $ ?: !. etc.).
• URLs
• Media (images, videos, and others).
• Links, hashtags, numbers, and English letters.
• Punctuation marks and diacritical marks.
• Line tap from the tweet text and spaces.
• Stop words
• Emojis

Algorithm 1 shows that the scraping process involves the initial step of authenticating access to the Twitter API.

**Algorithm 1:** Scraping data from Twitter.

1	Import twitter_api_module
2	Import *data_analysis_and_manipulation_module*
3	*twitter_api* = *twitter_api_module.authenticate_api()*
4	*spam_hashtags* ← [“#example”, “#sample”]
5	*spam_keywords* ← [“keyword1”, “keyword2”]
6	*spam_accounts* ← [“@account1”, “@account2”, …., “@accountn”]
7	*collected_spam_tweets* ← []
8	For account in *spam_accounts*
9	*tweets* ← *twitter_api.get_tweets_from_account(account)*
10	*retrieved_spam_tweet* ← []
11	For hashtag, keyword in *zip(spam_hashtags, spam_keywords)*
12	if hashtag in *tweets* or keyword in *tweets*
13	*retrieved_spam_tweet* ← *tweets*
14	*collected_spam_tweets* ← *drop_duplicates(retrieved_spam_tweet)*
15	*processed_spam_tweets* ← *drop_irrelevant (collected_spam_tweets)*
16	*ham_accounts* ← [“@AlArabiya”, ”@AlHadath”, “@emaratalyoum”, “@skynewsarabia”]
17	*ham_tweet* ← []
18	For account in *ham_accounts*
19	*ham_tweets* ← *twitter_api.get_tweets_from_account(account, since=Jan2021, until=March2021)*
20	*collected_ham_tweets* ← *drop_duplicates(ham_tweets)*
21	*processed_ham_tweets* ← *drop_irrelevant (collected_ham_tweets)*
22	*data_analysis_and_manipulation_module_to_csv(processed_spam_tweets*, “spam*_tweets.csv*”*)*
23	*data_analysis_and_manipulation_module_to_csv(processed_ham_tweets*, “ham*_tweets.csv*”*)*

In order to delve deeper into the data and ascertain the prevailing topic within the spam dataset, a technique called topic modeling was employed. Topic modeling, a widely recognized method in text analysis, finds its application in various domains, including text classification [11]. Bertopic [12] was employed for this data analysis stage, a neural topic modeling approach incorporating a class-based TF-IDF procedure.

**Fig. 1:**
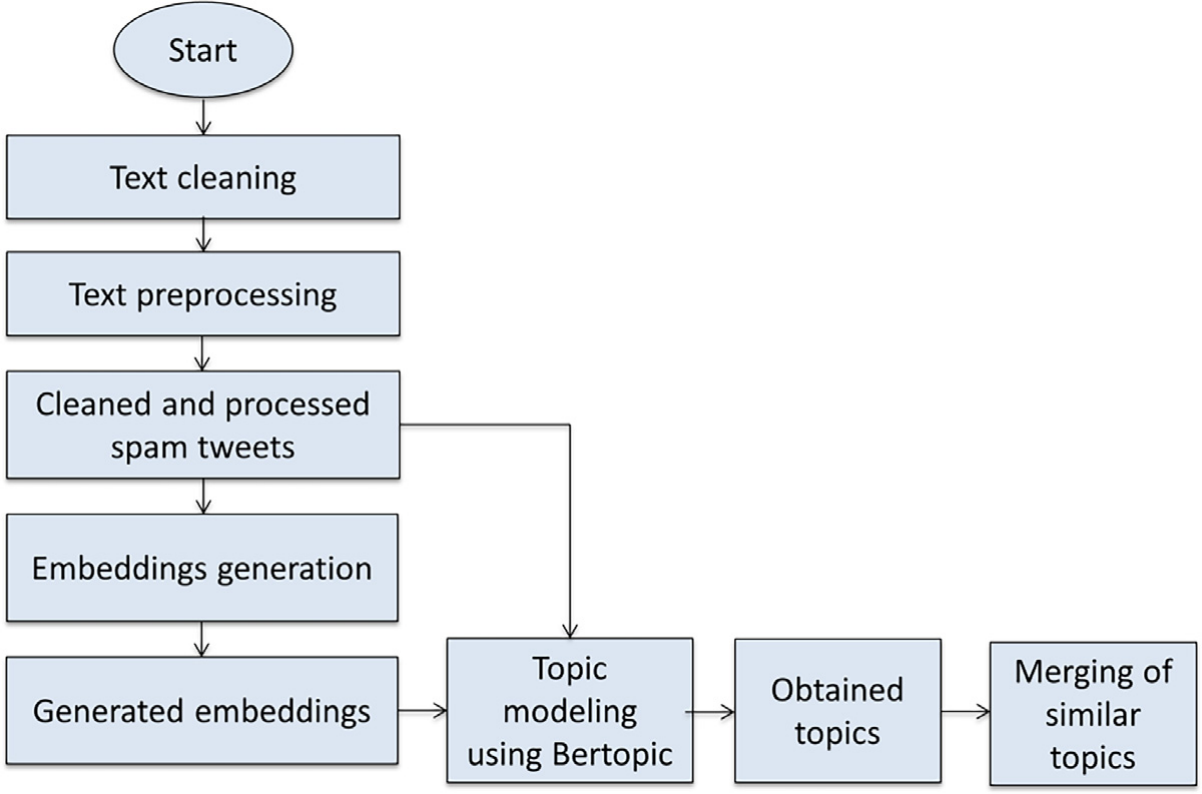
Illustration of topic modeling process.

The same spam tweets that are published in the dataset were used to perform topic modeling. As illustrated in Fig. 1, first, the spam tweets were cleaned by removing the Arabic stop words. After that, the cleaned spam tweets were preprocessed using the “aubmindlab/bert-large-arabertv02” [12] model of ArabertPreprocessor. Then, sentence embeddings were generated by applying the encoding method of the SentenceTransformer class and the “aubmindlab/bert-large-arabertv02” model from the sentence-transformers library to the processed Arabic spam tweets. Subsequently, the processed Arabic spam tweets and the generated embeddings were fed into the Bertopic model [13], which employed an encoding method to produce embeddings specifically tailored to the tweets. The Bertopic model parameters are specified below:

• language= “multilingual”
• n_gram_range= (1, 2)
• vectorizer_model= vectorizer_model
• nr_topics= 10
• min_topic_size= 5
• seed_topic_list= [[“الجنس”,“سكس”], [“شاهد الحذف”,“ كود خصم”], [“علاج”, “ضعف الانتصاب”],[“كلبه”], [“شهاده”, “شهادات”]]
• calculate_probabilities= True

Table 6 provides an overview of the topic distribution generated by the Bertopic model. The nature of spam tweets discussed in this article might entail the presence of unsuitable language and content inherent to the nature of such communication. Such words appear in Table 5. It is imperative to acknowledge that their inclusion is imperative, as they constitute the fundamental underpinning of the research subject elucidated in this discourse.

**Table 6:** Distribution of topics of the spam tweets.

Topics	Percentage	Topic Words and Weights	Dominant Words and their English Translation	Example and its Translation
Topic 0/Sex	81.3%	0.07137782844522596 سكس, الحذف 0.06747493866048158, 0.056819966375677156 راحتي, 0.05498130263158924 معصيتي, شاهد 0.05342414565893068	[سكس, قذف, جنسية, راحتي معصيتي] Sex, ejaculation, sexuality, my comfort is my disobedience]	أهلا وسهلا بكم ◻ علاج القذٕف السريع الامريكي الطبيعي علاج ضعف الانتصاب ◻ لطلب التواصل واتساب من هنا صفحة الاخصائية ام نايف Welcome to ◻ Rapid Ejaculation Treatment American Natural Treatment of Erectile Dysfunction ◻ To request WhatsApp communication from here, the page of the specialist Um Nayef
Topic 1/Education	11.9%	شهاده 0.19045250600174288, ايلتس 0.1536584290590755, معتمده 0.11179323181989126, شهادات 0.1080893375613558, جامع 0.1055176410644102	[شهاده,ايلتس,معتمده,شهادات,جامعه] [Certificate, IELTS, Accredited, Certificates, University]	ابشر الان يمكنك الحصول علي شهاده ايلتس معتمده و موثوقه و بجميع الضمانات فقط تواصل معنا Preach, now you can get an accredited and reliable IELTS certificate with all guarantees, just contact us
Topic 2/Trading	3.9%	فوركس 0.23409815221209496, التجاره 0.18003278392417968, التداول 0.17364889651197843, نظام 0.1655396312751765, التجاوب 0.1635110129138333	[فوركس,التجاره,التداول,نظام] [forex, trading, trading, system]	اي تحليل اضافي غير الموجود بالتوصيات تواصل خاص عملات ذهب نفط فوركس الذهب Any additional analysis that is not in the recommendations. Special communication. Gold, oil, forex, gold
Topic 3/Offers	2.9%	خصم 0.2733753948662136, كود 0.23992395951924303, كوبون 0.174867036670919, اند 0.1419925687751372, سيفي 0.13748501434660937	[خصم,كود,كوبون] [discount, code, coupon]	اكواد كود خصم نمشي كوبون سيفي كود ممزورلد مامزورلد هاي بوبي بيبي ستايلي فوغا كلوسيت سيتي ماكس فاشون فاشن قسيمة شراء موقع متجر تطبيق Namshi discount code codes, Sivvi coupon, Mumzworld code, Mumzworld Hibobi, Baby Styley, Voga, Closet, City, Max, Fashion, Fashion, purchase voucher, app store website

**Fig. 2:**
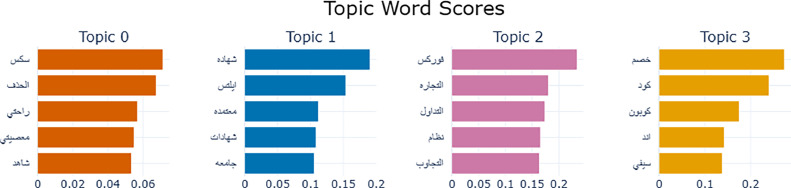
Generated topics using bertopic.

The obtained topics were visualized using the “visualize_barchart” method of the Bertopic model. Finally, similar topics were merged by applying the merge_topics() method of the Bertopic model. The get_topic_info() method was used to obtain the number of documents in each topic and compute the percentage of documents in each topic. Table 6 illustrates the distribution of the topics of the spam tweets*.* The top words in each topic were detected by utilizing the get_topic() of the Bertopic. The generated topics are illustrated in Fig. 2. The top words in each topic along with their weighted scores, are more clearly represented in Table 6.

Among the spam topics identified, the most dominant one was related to sex, accounting for a significant portion of the data, precisely 81.3 %. Following closely behind was the education topic, representing 11.9% of the data. The remaining spam instances predominantly revolved around advertising trading opportunities and offers. The first topic, sex, consists of sensitive terms like 'سكس', 'معصيتي', and 'راحتي', where 'سكس' word has the highest weight of 0.071. The second topic, Trading, has words like 'شهاده', 'ايلتس', 'معتمده', and 'شهادات' scoring a weight of 0.19, 0.154, 0.112, 0.108 respectively. In the third topic, the three most representative words are 'فوركس', 'التجاره', and 'التداول', recording weighted scores of 0.234, 0.18 and 0.174, respectively, which represent the Education Topic. Finally, the fourth topic, which stands for Offers Topic, has 'خصم', 'كود', and 'كوبون' words where 'خصم' records the highest score of 0.273.

When analyzing the available resources for identifying spam tweets, a notable inconsistency exists between the richness of English data and the absence of Arabic data. There are multiple sources available for English tweet spam detection data. Some of the publicly available sources in English are as follows:

1. The NSCLab has released datasets of spam tweets by the authors in [15].
2. In [16], the authors have collected context-specific spam datasets.
3. The authors in [17] have created UtkMl's Twitter Spam Detection Competition dataset, which the authors in [18] employed to develop an advanced spam detection model.
4. HSpam14 [19], a dataset, was assembled for spam research purposes.

Researchers have effectively utilized these datasets to propel advancements in spam detection techniques and algorithms. For example, the authors used the NSCLab data lately in [20]. In contrast, there is a noticeable lack of publicly accessible Arabic tweet spam detection data, as indicated in Table 7. Despite the increasing presence of Arabic content on social media platforms, the Arabic language still lacks the availability of a comprehensive dataset. The reasons for this scarcity may vary due to multiple factors, such as:

1. Geographical or Cultural Variation: Using dialectal language on social networks [21] has affected the presence of data that captures the variation in spam messages from different regions. Table 7 shows that most previous research has focused on collecting data related to specific regions.
2. Language Complexity: The linguistic characteristics of Arabic, including semantic and syntactic complexities [22], could pose challenges in constructing precisely annotated datasets.
3. Research Gap: Insufficient dedicated research in the Arabic spam detection domain might have resulted in the absence of concerted efforts to compile and release comprehensive datasets.

**Table 7:** Comparison with datasets in the literature.

Dataset	Size	Labeled	Spam Tweets Count/ (%)	Ham Tweets Count/(%)	Spam to ham ratio	Source	Hashtags Used	Spam Categories	Labelling Technique	Available online
[7]	102K	Yes	12K/11.7%	90K/ 88.3%	1:7.5	Middle East	Multiple	Advertising	Manual	Link not working
[8]	40K	Yes	12K/30%	28K/ 70%	1:2.3	Saudi Twitter	Multiple	Various Categories	Manual	No
[9]	313K	Partial	420/8.4%	4580/ 91.6%	1:10.9	Diverse Arabic	Single	Various Categories	Manual	No
[10]	2K	Yes	Not Given	Not Given	Not Given	Gulf Dialect	Multiple	Various Categories	Keywords	No
This Dataset	13.2K	Yes	2.2K/15.1%	11.2K/84.9%	1:5.6	Diverse Arabic	Multiple	Various Categories	Hybrid (Keywords & manual intervention)	Yes

The scarcity of Arabic tweet spam detection data carries substantial implications for research and advancements in this field. It hinders the ability of researchers to develop effective spam detection models tailored to the Arabic language. To address this challenge, researchers may need to explore alternative strategies like data augmentation, cross-lingual transfer learning, or creating annotated datasets.

Making the Arabic spam tweet and ham dataset publicly available is very important. Online spam occurs in various forms, including malware, posting commercial URLs, spreading fake news or abusive content, and automatically generating substantial content volumes [23]. Another side of online spamming involves increasingly utilizing machine learning models to generate counterfeit product reviews and services [24] or creating fake news, URLs, and advertisements. A study on Twitter shows that one out of every 21 tweets is considered spam, and approximately 15% of active users consist of autonomous agents, namely social bots [25]. This high percentage of spam on Twitter proves the importance of having datasets to build spam filtering algorithms and ban spam tweets.

A comparison between the data collected from Arabic spam and ham tweets, along with other datasets described in the relevant literature, is presented in Table 7. The table shows that the size of spam tweets is significantly smaller for all datasets when compared to ham tweets. The intentional choice of the proportion thus aims to imitate a real environment and facilitate the accurate use of spam classifiers. To illustrate, in social media networks, legitimate content typically holds a dominant presence. In addition to the sizes, the table also shows the spam-to-ham ratio, which refers to the proportion of spam-to-ham tweets in each dataset. For example, a spam-to-ham ratio of 1:7.5, as presented in the dataset [7], indicates that most tweets are legitimate, as there are 7.5 ham tweets in the data for one spam tweet. The dataset in [8] has 2.3 ham tweets for every collected spam tweet. So, the percentage of ham tweets is double the percentage of spam tweets. The dataset in [9] used only one single hashtag to collect all the data. So, the classifier will only fit this specific data. Some literature, such as [10], have built their spam detection on 2K tweets. Nevertheless, this data is considered small, and the model will not learn to differentiate between spam and ham tweets effectively.

The collected dataset in this research comprises different accounts and uses multiple hashtags to cover diverse spam categories, including advertising, inappropriate words, etc. Besides, unlike datasets presented in the literature, the dataset presented in this paper contains tweets from around the world and covers various writing styles for the Arabic language. The collected spam and ham tweets data is labeled thoroughly. The labeling procedure is based on a hybrid approach in order to avoid labeling mistakes by utilizing only keywords and human errors in manual labeling.

## Limitations

Not applicable.

## Ethics Statement

Participant data has been fully anonymized, and the platform(s)' data redistribution policies were complied with.

## CRediT authorship contribution statement

**Sanaa Kaddoura:** Data curation, Conceptualization, Methodology, Writing – review & editing. **Safaa Henno:** Data curation, Methodology, Software.

## Data Availability

Dataset of Arabic Spam and Ham Tweets (Original data) (Mendeley Data)

## References

[1] Kaddoura S., Henno S. (2023).

[2] Kaddoura S., Alex S.A., Itani M., Henno S., AlNashash A., Hemanth D.J. (2023). Arabic spam tweets classification using deep learning. Neural Comput. Appl..

[3] Al Arabiya. https://www.alarabiya.net/, 2021(Accessed 27 January 2021).

[4] Al Hadath. https://www.alhadath.net/, 2021(Accessed 28 January 2021).

[5] Emarat Alyoum. https://www.emaratalyoum.com/, 2021(Accessed 29 January 2021).

[6] Sky News Arabia. https://www.skynewsarabia.com/, 2021(Accessed 30 January 2021).

[7] Mubarak H., Abdelali A., Hassan S., Darwish K. (2020). International Conference on Social Informatics.

[8] Balfagih A., Keselj V., Taylor S. (2022). Proceedings of the 6th International Conference on Information System and Data Mining.

[9] Alkadri A.M., Elkorany A., Ahmed C. (2022). Enhancing detection of Arabic social spam using data augmentation and machine learning. Appl. Sci..

[10] Alorini D., Rawat & D.B. (2019). 2019 International Conference on Computing, Networking and Communications (ICNC).

[11] Anupriya P., Karpagavalli S. (2015). International Conference on Advanced Computing and Communication Systems. IEEE.

[12] W. Antoun, F. Baly, H. Hajj, Arabert: Transformer-based model for arabic language understanding. arXiv preprint arXiv:2003.00104.‏ (202)

[13] M. Grootendorst, BERTopic: Neural topic modeling with a class-based TF-IDF procedure. arXiv preprint arXiv:2203.05794.‏ (2022)

[14] Digital Commons Data, Dataset Archiving. https://data.mendeley.com/archive-process

[15] Chen C., Zhang J., Chen X., Xiang Y., Zhou W. (2015). 2015 IE*EE International Conference on Communications (ICC)*.

[16] Kawintiranon K., Singh L., Budak C. (2022). Traditional and context-specific spam detection in low resource settings. Mach. Learn..

[17] Bhidya M. (2019). https://Kaggle.Com/Competitions/Twitter-Spam.

[18] Liu X., Lu H., Nayak A. (2021). A spam transformer model for SMS spam detection. IEEE Access.

[19] Sedhai S., Sun A. (2015). Proceedings of the 38th International ACM SIGIR Conference on Research and Development in Information Retrieval, ACM Conferences.

[20] Kumar C., Bharti T.S., Prakash S. (2023). A hybrid data-driven framework for SPAM detection in online social network. Procedia Comput. Sci..

[21] Kaddoura S., Itani M., Roast C. (2021). Analyzing the effect of negation in sentiment polarity of facebook dialectal Arabic text. Appl. Sci..

[22] Kaddoura S., Ahmed R.D., D J.H. (2022). A comprehensive review on Arabic word sense disambiguation for Natural Language Processing Applications. WIREs Data Min. Knowl. Discov..

[23] Varol O., Ferrara E., Davis C., Menczer F., Flammini A. (2017). Proceedings of the International AAAI Conference on Web and Social Media.

[24] Yao Y., Viswanath B., Cryan J., Zheng H., Zhao B.Y. (2017). Proceedings of the 2017 ACM SIGSAC Conference on Computer and Communications Security.

[25] Inuwa-Dutse I., Liptrott M., Korkontzelos I. (2018). Detection of spam-posting accounts on Twitter. Neurocomputing.

